# Editorial: Novel targets for ovarian cancer stem cells

**DOI:** 10.3389/fonc.2023.1325066

**Published:** 2023-11-01

**Authors:** Yinu Wang, Livia E. Sima, Dongyu Jia, Guangyuan Zhao, Yaqi Zhang, Jia Xie

**Affiliations:** ^1^ Department of Obstetrics and Gynecology, Feinberg School of Medicine, Northwestern University, Chicago, IL, United States; ^2^ Department of Molecular Cell Biology, Institute of Biochemistry of the Romanian Academy, Bucharest, Romania; ^3^ Department of Molecular and Cellular Biology, Kennesaw State University, Kennesaw, GA, United States; ^4^ Department of Radiation Oncology, St. Jude Children’s Research Hospital, Memphis, TN, United States

**Keywords:** ovarian cancer, cancer stem cells, novel targets, prognostic indicators, epigenetic

Although ovarian cancer (OC) has been considered as a chemo-sensitive tumor type, the majority of patients develop chemoresistance, which drives tumor relapse within two years. Cumulative evidence supports that the presence of cancer stem cells (CSCs), in tumor residuals are responsible for driving chemoresistance and tumor relapse. CSCs represent a rare population of malignant cells displaying upregulated expression of “stemness”-associated transcription factors and enhanced self-renewal and tumor initiation capability. Given the pivotal role of CSCs in regulating chemoresistance and tumor recurrence, it is urgent to develop novel strategies to eradicate ovarian CSCs (OCSCs) and prevent tumor relapse. This Research Topic gathers 2 original articles, 2 reviews and 1 systematic review on the involvement of CSCs in OC progression, analysis of impact of stemness markers expression on patient’s prognosis and new strategies to target OC that could be translated therapeutically.

The first work contained in this topic (Xie et al.) highlights the role of the stem cell transcription factor OCT4 in OC. Authors underline the impact of abnormal expression of this marker in adult cancer cells undergoing reprogramming upon transformation. Using immunohistochemistry (IHC) and tumor microarrays (TMAs), they revealed that OCT4 is upregulated in OC samples and metastatic tissues, as compared to ovarian benign cyst tissues. Moreover, OCT4 was found to function through the PI3K/AKT/mTOR signaling axis and to increase the EMT in cancer cell lines.

In a similar approach, the second article (Xiu et al.) describes the role of retinoic acid receptor gamma (RARG), which is usually selectively expressed in hematopoietic stem cells. However, instead of TMAs, authors used information stored in TCGA and GTEx databases to emphasize the relevance of RARG in the context of OC. Increased expression of RARG was found in OC samples as compared to normal ovarian tissue. Next, IHC and RT-qPCR analyses of OC tissues have confirmed the presence of this stem cell marker in tumors at gene and protein expression level. Both OCT4 (Xie et al.) and RARG (Xiu et al.) expression is highly regulated in OC tumors, which supports the uncontrolled proliferation of cancer cells and decreases the chances of patient survival if highly expressed. Knockdown of either of the two genes in OC cells decreases the aggressive potential of OC cells. These proteins could serve as potential molecular targets, as well as diagnostic biomarkers for personalized treatment in OC.

Next, the first review (Wilczynski et al.) introduces a novel therapeutic regimen named the “Dynamic PHarmacologic survEillaNCE” (“DEPHENCE”) system that could translate into an alternative treatment scheme in addition to the current options available for high-grade serous ovarian cancer (HGSOC) patients. Importantly, authors provide an inventory of markers used for characterization of OCSCs, and further elaborate their function, and clinical significance as well as the signaling pathways involved in the stem cell phenotype. Moreover, the elements of the OC metastases tumor microenvironment are listed in detail and correlated to relevant clinical features. These molecular and cellular characteristics help define different HGSOC types that could be targeted as individual entities with specific features. They have detailed the bases of the DEPHENCE approach principles with the expressed long-term goal to obtain individual patient’s cure. One of the most important stated principle is that “every line of treatment should simultaneously target cancer cells, OCSCs, and elements of TME, as well as generate potentialization of the patient’s immune status”. More than a therapeutic strategy, the DEPHENCE system is a philosophy that should guide oncologists to make the most informed diagnostic and therapeutic decisions for the OC patients.

This Research Topic is focusing on defining novel biomarkers and therapeutical targets for OCSCs, but not limited to. Recently, inflammation has been well-demonstrated to be responsible for driving tumor development and recurrence. Here, we appreciate the systematic study on the OC tumor cohorts reported in the Web of Science, PubMed, Cochrane library, Embase, and China National Knowledge Infrastructure (CNKI) (Mao and Yang), which performed the meta-analysis of the prognostic role of systemic immune-inflammation index (SII) in predicting OC. This study significantly enhances the clinical relevance of this topic, and emphasizes the importance of a blood test-based systemic immune inflammation index for predicting OC prognosis. This systematic meta-analysis involves 1546 patients, and assesses the prognostic value of SII for overall survival (OS) and progression free survival (PFS) in patients with OC based on the hazard ratios (HRs) and corresponding 95% confidence intervals (CIs). The analysis results confirmed that a high SII is correlated with poor OS and PFS in OC patients, indicating an independent prognostic indicator for OC diagnostic.

Interestingly, this Topic also includes a review article (Fu et al.), which comprehensively discusses the function of another potential indicator - LINE-1 - for the diagnosis, and prognosis of OC and other gynecologic malignancies. LINE-1, a non-long terminal repeat (non-LTR) retrotransposon, that is still active in humans, its activity is dysregulated in disease, as their expression is usually epigenetically repressed. LINE-1 hypomethylation resulting in genomic instability, is closely associated with the development of various malignancies, and is expected to be an independent biomarker of early diagnosis and poor prognosis of tumors, including OC. This review thoroughly discussed mechanisms involved in the oncogenic functions of LINE, including LINE-1 insertion dependent proto-oncogenes activation, overexpression of LINE mRNA, LINE-1 promoter hypomethylation, and overexpression of LINE-1 reverse transcriptional productions. Evidence supports that LINE-1 hypomethylation is an early event in the development of EOC and a tumor-specific LINE-1 insertion is associated with chemoresistance, suggesting its independent role as a potential indicator for prognosis of poor gynecological malignancies. Importantly, this review also summarized current epigenetic therapies, such as DNA methyltransferase inhibitors (DNMTis), and histone demethylase inhibitors, have been explored and evidenced to inhibit LINE-1 mRNA expression thereby preventing tumor progression.

All in all, we are confident that the audience of this Research Topic will discover relevant new concepts and new evidence on the involvement of OCSCs in the OC tumor progression and how this cancer cell subset could be targeted in potential new therapeutic clinical interventions.

## Author contributions

YW: Writing – original draft, Writing – review & editing. LS: Writing – original draft, Writing – review & editing. DJ: Writing – review & editing. GZ: Writing – review & editing. YZ: Writing – review & editing. JX: Writing – review & editing.

